# On Sympathetic Ophthalmia

**Published:** 1875-08

**Authors:** 


### On Sympathetic Ophthalmia.

In the *Berliner Klinishche Wochenschrift*, April 5th, will be found a concise historical sketch, by Dr. Grossman, of the phenomena which make up what is now termed sympathetic ophthalmia. According to this writer, the nature of the affection was first recognized by the Vienna School in the early part of this century, although it was Mackenzie who in 1844 first described a variety of sympathetic iritis. The writings of later surgeons, and especially those of Von Græfe and Mooren, agree in the doctrine that the starting-point of the disturbance in the second eye is to be found in the presence of cyclitis in the eye originally injured or affected. In accordance with this, Dr. Grossman gives a very complete clinical sketch of the symptoms of cyclitis, and enumerates at length the many circumstances under which it may take its origin, such as injuries of all kinds, with or without any external wound; also certain intraocular causes, such as tumors detachment of the retina, anterior synechia, extensive posterior synechia, and the varieties of anterior staphyloma, most of which are not generally recognized as possible causes. As regards treatment, when once any structural alteration has taken place in the second eye, the removal of the offending eye must be immediately carried out. If performed sufficiently early, Grossman has found that iridectomy will be found of great use to the second eye, provided it be completely executed and to a considerable extent; otherwise it will prove of no service; and if its performance be delayed till the iris has contracted adhesions, or has become entensively altered in texture, the operation will be impracticable owing to the impossibility of removing any of the iris. and the probable result of such unsuccessful interference will be degeneration and atrophy of the entire globe. Should, however, the iridectomy be performed early enough, in the course of time, it may be of many months, the eye may regain some considerable amount of useful vision, although it is probable that extraction of the lens will have to be undertaken to bring about even this partial recovery. From many cases of the kind Dr. Grossman chooses, as an illustration of his remarks, the case of a man, aged twenty-three, who was struck in the left eye with a small splinter of iron; for this injury the man was under treatment for nine months, at the end of which time he resumed work, but was compelled to discontinue it at the end of a week in consequence of the ciliary irritation which made its appearance in the right eye. These symptoms rapidly ripened into those of iritis, and he now came for the first time under the author's care. The left eye, which was blind, shrunken, and painful, was removed, and at once the symptoms in the other eye were alleviated; at the end of a month a large iridectomy was successfully performed, and a steady and still further improvement took place; in the result, the natural and healthy appearance was restored, and the patient regained very useful vision=$\frac{2}{3}$. Examination of the enucleated eye revealed a splinter of iron embedded in the ciliary region. Dr. Grossman insists on the importance of publishing such cases in great detail, in order that those members of the profession who are not practising ophthalmic surgery may be sufficiently alive to the danger and to the insiduous nature of this form of ophthalmia.—*London Medical Record, May* 19, 1875.—*Monthly Abstract Med. Science.*

